# Biliary Ascariasis in a Patient with Choledochoduodenal Fistula

**DOI:** 10.4269/ajtmh.25-0429

**Published:** 2025-09-04

**Authors:** Ravi Teja Reddy, Venkatesh Vaithiyam, Sanjeev Sachdeva

**Affiliations:** Department of Gastroenterology, GB Pant Hospital and Associated Maulana Azad Medical College, New Delhi, India

**Figure 1.**
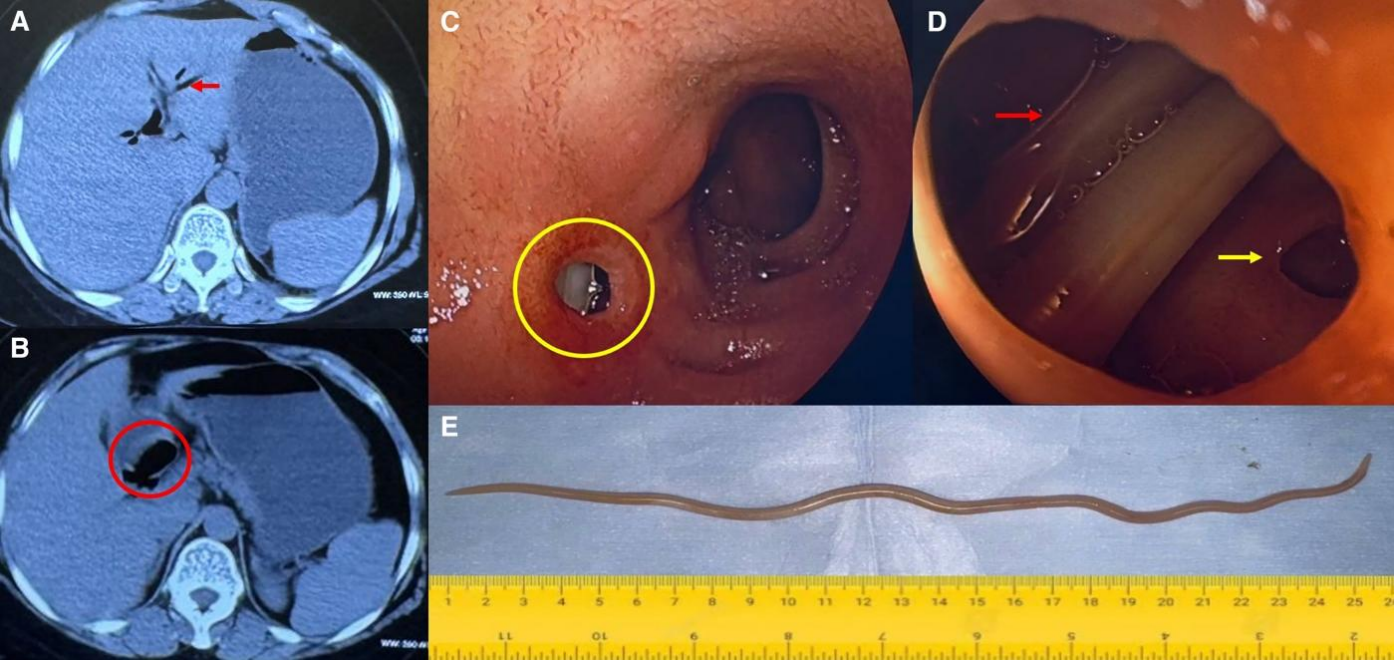
Computed tomography images (**A**) shows central and peripheral pneumobila (red arrow); (**B**) Air filled common bile duct adjacent first part of duodenum (red circle), suspicious of communication between them; (**C**) Upper Gastrointestinal endoscopy shows a fistulous opening in the anterior wall of the first part of the duodenum (yellow circle) with a worm inside the fistula; (**D**) Near view of the fistula shows *Ascaris lumbricoides* (red arrow) and biliary radicals (yellow arrow); (**E**) *Ascaris lumbricoides* retrieved from the fistula.

A 54-year-old woman presented with a history of dull aching pain in the upper abdomen and occasional non-bilious vomiting over the past month. There was no history of fever, jaundice, weight loss, or changes in bowel habits. She had undergone abdominal surgery several years previously for symptomatic gallstone disease. Physical examination revealed a right subcostal surgical scar. Laboratory investigations showed haemoglobin of 9.8 g/dL, total bilirubin of 1.5 mg/dL, aspartate aminotransferase of 78 IU/L, alanine aminotransferase of 84 IU/L, and alkaline phosphatase of 387 IU/L. The peripheral smear demonstrated microcytic hypochromic anaemia, and serum iron and ferritin levels were 20 *µ*/dL and 15 ng/mL, respectively. The absolute eosinophil count was 1350/*µ*L. Computed tomography (CT) indicated a post-cholecystectomy status and suspected communication between the retro-duodenal portion of the common bile duct and the first part of the duodenum, with extensive pneumobilia observed in the CBD and intrahepatic ducts (Figure 1A and B). These findings were consistent with a choledochoduodenal fistula (CDF). In view of the abdominal pain and CT Findings, an upper gastrointestinal endoscopy was performed, which revealed a small opening in the first part of the duodenum and a worm was seen through it (Figure 1C and D). The worm was retrieved using alligator forceps, and gross examination revealed that it was an adult *Ascaris lumbricoides *(Figure 1E). The patient was started on albendazole and iron supplements, and her symptoms improved.

The natural habitat of *Ascaris lumbricoides* is the jejunum, and it seeks and migrates into small orifices, such as the ampulla^1^. Biliary ascaris is usually asymptomatic, however blockage of the ampulla by dead or live worms can cause symptoms^2^. This case illustrates the uncommon but reported complication of biliary ascariasis passing through CDF in a post-cholecystectomy patient^3^. Patients with prior biliary surgery, cholecystectomy, choledocholithotomy, endoscopic sphincterotomy, and pregnant women have an increased risk of biliary ascariasis^4^^,^^5^. The endoscopic view of a live worm emerging from a fistula is characteristic of this condition. Endoscopic worm removal is a safe and effective treatment. Antiparasitic drugs, such as albendazole or mebendazole, are necessary to prevent recurrence.
